# Supplementary material for: A new automatic sugarcane seed cutting machine based on internet of things technology and RGB color sensor
Source: PLoS One. 2024 Mar 28;19(3):e0301294. doi: 10.1371/journal.pone.0301294 (PMC10977673; doi:10.1371/journal.pone.0301294)
Supplement: Attachment — Submitted filename: ID PONE-D-24-05665 Reviewer.doc [file pone.0301294.s001.doc]

**Comment on Manuscript**

Manuscript Number: **PONE-D-24-05665**

Full Title: **A New Automatic Sugarcane Seed Cutting Machine Based on Internet of Things Technology**

*Submitted to* ***PLOS ONE*** *(SCIE, IF = 3.7)*

The pulse widths (PW) of three color channels (Red-R, Green-G, and Blue-B) of the sensors under the laboratory conditions have been proposed in this study. The IoT and RGB color sensors are possible to get the analytical indicators similar to those achieved with other automatic systems for cutting sugar cane seeds without requiring using computers. This study is interesting however there are some drawbacks that the authors should address them to improve this study.

1. In the introduction, the contributions of this study must be provided clearly especially the proposed methods comparing with the existing studies.
2. At item 2.3, the authors stated that “The operational procedure for cutting sugarcane seeds is displayed in Figure 4.” This statement is inaccurate to describe this content.
3. In table 2, the authors compared with other technologies, based on the certain values, this technology is not benefit than previous technologies.
4. The scientific basis of combined methods between the internet of things (IoTs) and RGB sensors must be provided.
5. Its significance of this study is inadequate in the novelty methods.
6. The limitation of this study is not provided as well as its application in the food manufacturing.
